# Depression-related innate immune genes and pan-cancer gene analysis and validation

**DOI:** 10.3389/fgene.2024.1521238

**Published:** 2025-01-10

**Authors:** Yakun Yang, Wei Han, Xiaoyu Zhang, Hao Yuan, Ran Wang, Jia Yang, Cuixia An, Dongyang Huang

**Affiliations:** ^1^ Department of Pharmacology, The Key Laboratory of Neural and Vascular Biology, The Key Laboratory of New Drug Pharmacology and Toxicology, Ministry of Education, Collaborative Innovation Center of Hebei Province for Mechanism, Diagnosis and Treatment of Neuropsychiatric Diseases, Hebei Medical University, Shijiazhuang, Hebei, China; ^2^ Sharing Service Platform for Large-Scale Scientific Research Instruments and Equipment, Hebei Medical University, Shijiazhuang, Hebei, China; ^3^ Central Sterile Supply Department, Baoding No. 1 Central Hospital, Baoding, Hebei, China; ^4^ Department of Psychiatry, The First Hospital of Hebei Medical University, Shijiazhuang, Hebei, China; ^5^ Operating Room, Cheng’an County People’s Hospital, Handan, Hebei, China

**Keywords:** depression, pan-cancer, GEO, WGCNA, machine learning, DEGs, immunoinfiltration

## Abstract

**Background:**

Depression, a prevalent chronic mental disorder, presents complexities and treatment challenges that drive researchers to seek new, precise therapeutic targets. Additionally, the potential connection between depression and cancer has garnered significant attention.

**Methods:**

This study analyzed depression-related gene expression data from the GEO database. Using data normalization, differential expression analysis, WGCNA, and machine learning, we identified core genes strongly associated with depression. These genes were validated in depression patients through q-PCR and examined for expression patterns and potential roles across various cancers.

**Results:**

We identified six core genes (GRB10, TDRD9, BCL7A, GPR18, KLRG1, and THEM4) significantly associated with depression and cancer. In depression, GRB10 and TDRD9, involved in cell growth and stress responses, exhibited elevated expression, while BCL7A, GPR18, KLRG1, and THEM4, linked to immune regulation and apoptosis, showed reduced expression, suggesting dysregulated cellular signaling and impaired immune function. In cancer, these genes displayed altered expression patterns across tumor types, influencing tumor progression, prognosis, and immune microenvironment modulation. Shared molecular pathways, such as immune dysregulation and apoptosis, highlight their potential as biomarkers and therapeutic targets for both depression and cancer.

**Conclusion:**

This study integrates bioinformatics and machine learning to uncover key molecular pathways and targets for depression, introducing innovative therapeutic prospects that may enhance precision treatment for depression. Furthermore, by revealing shared mechanisms between depression and cancer, we have identified six core genes with significant functional roles in immune regulation, apoptosis, and cellular signaling. These findings not only deepen our understanding of the molecular overlap between these conditions but also lay the groundwork for developing dual-targeted therapeutic strategies. This study uniquely contributes to bridging mental health and oncology research, offering new insights and hope for improving patient outcomes in both fields.

## 1 Introduction

As the pace of modern life accelerates, the rising prevalence of depression has been rising globally. Currently, there are approximately 3 billion people worldwide suffering from depression, making it one of the most prevalent mental disorders globally (World Health Organization, 2023). By 2030, depression is projected to become a leading cause of impaired quality of life (Ferrari et al., 2013; Whiteford et al., 2013). The primary clinical symptoms include anxiety, insomnia, tension, melancholy, lack of concentration, and in extreme cases, suicidal thoughts (Fried, 2017). Presently, pharmacotherapy and psychotherapy are the main approaches to treating depression (Farah et al., 2016). However, current efforts to identify reliable biomarkers for depression remain limited by challenges such as the heterogeneity of depressive disorders, insufficient sample sizes in studies, and a lack of integrated analysis across datasets. These limitations hinder the development of precise diagnostic and therapeutic strategies, underscoring the need for novel approaches to identify robust molecular markers. Furthermore, the absence of precise biomarkers and therapeutic targets for depression has led to significant challenges, such as the development of drug resistance and high relapse rates in patients undergoing long-term antidepressant therapy (Pompili et al., 2013; Barth et al., 2016; Cipriani et al., 2018). Additionally, prolonged use of antidepressants is associated with metabolic disorders and an increased risk of cardiovascular diseases (Segraves, 2007; Bostwick, 2010; Carvalho et al., 2016). Therefore, the identification of core genes and molecular markers closely associated with depression is essential to advance our understanding of its pathogenesis and improve early detection and treatment.

Recent studies suggest that depression and cancer, though distinct in clinical presentation, may share underlying molecular mechanisms such as immune dysregulation, chronic inflammation, and altered cellular signaling. These shared pathways not only highlight biological connections between the two conditions but also present an opportunity for integrated research approaches to uncover novel therapeutic targets. Cancer is one of the leading causes of mortality worldwide (Sung et al., 2021), with the most common types being breast, lung, colon, rectal, and prostate cancers (World Health Organization, 2020). Numerous studies have established a significant positive correlation between cancer and mental disorders (Aass et al., 1997; Ford et al., 1995). Specifically, around 25% of cancer patients exhibit depressive symptoms at the early stages of their illness, with this percentage rising dramatically to 77% in the advanced stages, indicating a high prevalence of severe depression (Bukberg et al., 1984). Exploring shared genes between depression and cancer is scientifically significant, as these conditions may involve common biological pathways such as immune dysregulation and chronic inflammation, which could reveal new therapeutic targets for both diseases.

In this study, we employed bioinformatics and machine learning techniques to analyze the correlation between depression and pan-cancer, identifying core genes shared between the two conditions. This research provides new perspectives for future therapeutic strategies and lays the foundation for targeted treatments based on shared mechanisms between these diseases. Despite these advances, current research largely focuses on either depression or cancer independently, with limited studies investigating the genetic and molecular intersections of these conditions. Moreover, most studies rely heavily on gene expression data without integrating advanced computational approaches to identify and validate core molecular markers. Shared immune pathways, such as chronic inflammation, immune dysregulation, and alterations in cellular signaling, have been implicated in both depression and cancer, highlighting overlapping molecular mechanisms that may influence disease progression and therapeutic responses. Understanding these pathways could provide critical insights into how immune function contributes to the pathophysiology of both conditions. This gap in knowledge underscores the need for systematic research to elucidate shared mechanisms and identify potential therapeutic targets. By employing bioinformatics and machine learning techniques, our study provides a comprehensive analysis of the shared genetic basis of depression and pan-cancer, offering insights into novel pathways and molecular targets that may lead to more precise diagnostic and therapeutic strategies.

## 2 Methods and materials

### 2.1 Download and processing of depression datasets

The GSE76826 dataset, derived from blood cell samples, and the GSE98793 dataset, derived from whole blood samples, were downloaded from the Gene Expression Omnibus (GEO), accessible at https://www.ncbi.nlm.nih.gov/geo. The collected datasets are summarized in Supplementary Table S1. To analyze changes in mRNA expression, we conducted a differential expression analysis of depression-related genes using the “limma” package in the R environment (version 4.3.0), with access to the package at https://www.bioconductor.org/packages/release/bioc/html/limma.html (accessed 13 April 2024). Data normalization was performed using the “normalizeBetweenArrays” function from the “limma” package, employing quantile normalization to ensure comparability between samples. Differentially expressed genes (DEGs) were identified based on a p-value <0.05, a false discovery rate (FDR) <0.05, and a log2 fold change (|log2FC|) > 0.5. The p-value <0.05 and FDR <0.05 thresholds were chosen to ensure statistical rigor, minimizing false positives in the identification of significant DEGs, while the |log2FC| > 0.5 criterion captures genes with moderate but potentially biologically significant changes in expression. These thresholds align with widely accepted standards in transcriptomic studies, balancing the trade-off between sensitivity and specificity, particularly in studies with moderate sample sizes. A volcano plot was generated to visualize these results (Ritchie et al., 2015). Subsequently, we used the “pheatmap” package in R to create a heatmap of the selected differentially expressed genes (DEGs) (https://cran.r-project.org/web/packages/pheatmap/index.html, accessed 13 April 2024). The goal of this analysis was to identify key genes associated with depression, laying the foundation for subsequent functional and pathway analyses.

### 2.2 Functional enrichment analysis of DEGs

Functional enrichment analysis was performed on the identified DEGs. A commonly used technique for assigning function to genes is Gene Ontology (GO), which includes molecular function (MF), biological process (BP), and cellular component (CC) categories. KEGG enrichment analysis was conducted to identify pathways associated with the DEGs. GO functions and KEGG pathways were analyzed using the “GOplot” package and the “clusterProfiler” package in R (Walter et al., 2015).

### 2.3 Identification of depression-related key gene modules via WGCNA

Weighted Gene Co-expression Network Analysis (WGCNA) was performed using the “WGCNA” package in R (version 4.3.0) (https://cran.r-project.org/web/packages/WGCNA/index.html, accessed 12 January 2024) (Langfelder and Horvath, 2008). Genes were modularized through WGCNA, with non-clustering genes filtered out, and the remaining genes used to construct a co-expression network. This module was analyzed using dynamic tree cutting and hierarchical clustering. Module eigengenes and clinical traits were correlated using module membership (MM) and gene significance (GS). Hub modules with an absolute MM value of 0.05 were considered highly correlated. Modules with MM > 0.8 and GS > 0.2 indicated high connectivity and clinical relevance.

### 2.4 Identification of core genes via machine learning

To identify core genes associated with depression, we employed machine learning techniques, including Support Vector Machine-Recursive Feature Elimination (SVM-RFE), LASSO regression, and Random Forest (RF) analysis.

In the SVM-RFE model, features were ranked iteratively based on their contribution to classification performance, and the subset with the highest accuracy was selected. Parameters were tuned using strict ten-fold cross-validation, optimizing the penalty parameter (C) within a range of 0.01–100 to minimize classification error and improve generalization. The final C value was determined as 1 (Huang et al., 2014).

LASSO regression, performed using the “glmnet” package in R, was used to compute, select, and retain relevant variables. The lambda parameter was optimized through ten-fold cross-validation using the “1 SE” criterion, which selects the simplest model with performance within one standard error of the minimum deviance. Binomial distribution was applied to classify variables, and variables with non-zero coefficients after shrinkage were selected (Ishwaran and Kogalur, 2010).

For the Random Forest model, 500 trees were constructed, and the number of features randomly selected at each split (mtry) was set to the square root of the total features. The relative importance score of each gene was calculated based on its contribution to reducing the Gini impurity across all trees. Genes with relative importance scores above 0.25 were identified as critical determinants.

These three machine learning approaches were used in combination to screen for essential core genes, ensuring a robust and comprehensive identification process.

### 2.5 Real-time quantitative RT-PCR

Our study involved blood samples from six patients diagnosed with depression and eight healthy controls. The study protocol strictly adhered to the principles outlined in the Declaration of Helsinki to ensure the ethical handling of human tissues. Additionally, the study was approved by the Clinical Research Ethics Committee of the First Hospital of Hebei Medical University (NO. 20210742). Written informed consent was obtained from each participant after providing them with detailed information about the study’s purpose, procedures, and potential risks and benefits. Participants were assured that their involvement was voluntary, and they could withdraw at any time without repercussions.

To protect privacy and ensure confidentiality, all samples were assigned unique anonymized codes during collection. Identifiable personal information was securely stored and accessible only to authorized personnel for essential purposes. Data used in the analysis were completely de-identified, and all results were reported in aggregate form to prevent re-identification of individuals.

Total RNA was extracted from human blood using an RNA isolation kit (RNAiso, Takara, San Jose, CA, United States). The isolated RNA was dissolved in 20 μL of DEPC-treated water and used for reverse transcription with the PrimeScript™ RT reagent Kit with gDNA Eraser (Takara) and a thermal cycler (Eppendorf, Hamburg, Germany). The resulting cDNA was used for quantitative PCR (qPCR) detection, with amplification curves generated using SYBR Premix Ex Taq II (Takara). Primers were designed based on sequences from NCBI and optimized for specificity and efficiency. These primers were chosen to target conserved regions of the core genes identified in the study to ensure accurate quantification of expression levels. Details of the primers used are listed below. Statistical analysis of the qPCR data was conducted using GraphPad Prism (version 9.0). Relative expression levels of the core genes were calculated using the 2^−ΔΔCt method, and statistical comparisons between groups were performed using an unpaired two-tailed *t*-test. A p-value <0.05 was considered statistically significant. Such experimental validation ensured the accuracy and reliability of the gene expression levels identified, thereby reinforcing the robustness of our findings. The primers used for qPCR were:

GAPDH-Home-F: GGA​GCG​AGA​TCC​CTC​CAA​AAT.

GAPDH-Home-R: GGC​TGT​TGT​CAT​ACT​TCT​CAT​GG.

BCL7A-Homo-F: CAG​ATG​CCT​CCC​CCA​TCA​AA.

BCL7A-Homo-R: ACT​CCT​TCC​AAA​TCC​TGA​GAG​A.

GPR18-Homo-F: ACA​TCC​AAA​ATC​TTG​ATC​AGT​TA.

GPR18-Homo-R: AGG​ACA​GAC​TTT​CAA​AAT​GTT​T.

KLRG1-Homo-F: TTG​GGC​TGT​TTC​CTC​ACT​GAT.

KLRG1-Homo-R: TGG​AGT​AGT​TGG​AGC​CCT​GG.

THEM4-Homo-F: TCC​AGG​TTA​GAT​GCT​GCA​CA.

THEM4-Homo-R: GAG​TGG​AGC​TGG​CAA​ATT​G.

GRB10-Homo-F: CAG​CTT​TTG​CAG​GAA​CCC​AG.

GRB10-Homo-R: CTT​TAT​GCA​GAG​CCC​GTG​GT.

TDRD9-Homo-F: AAG​ACG​GTG​ACC​AAT​GTG​GAG.

TDRD9-Homo-R: CAA​AGA​TGG​CCT​TGG​ACC​TGG.

### 2.6 Construction of the PPI network

The protein-protein interaction (PPI) network for the core genes was constructed using GeneMANIA (http://www.genemania.org), a tool that predicts gene-gene interactions based on physical interactions, co-expression, co-localization, gene enrichment analysis, genetic interactions, and pathway predictions.

### 2.7 Development of a depression recurrence prediction model and core gene evaluation

After identifying core genes associated with depression, we constructed a composite nomogram model using the “rms” package in R (version 4.3.0) to predict depression recurrence. This model visually represents how different clinical factors contribute to the risk of recurrence. The “score” represents the weighted value of an individual clinical factor, while the “total score” represents the cumulative weight of all considered factors. The model’s reliability and predictive power were rigorously assessed using calibration curves. Decision curve analysis (DCA) and clinical impact curves were employed to evaluate the model’s clinical utility. Additionally, the diagnostic utility of key genes was evaluated by generating a receiver operating characteristic (ROC) curve using the pROC function in R, with the area under curve (AUC) serving as the metric.

### 2.8 Core gene expression and prognosis in pan-cancer

To explore the relationship between depression and cancer, we downloaded RNA sequencing and clinical data from The Cancer Genome Atlas (TCGA) database for 33 different cancer types. Data on normal tissue expression was obtained from the GTEx database. This step was crucial for verifying whether the core genes significant in depression could influence cancer. Expression levels of key genes were compared between case and control groups to identify any significant differences. For prognostic analysis, a univariate Cox regression analysis was performed using the “forestplot” R package, and forest plots were used to display the p-values, hazard ratios, and 95% confidence intervals.

### 2.9 Immune infiltration analysis of core genes in pan-cancer

The EPIC method was used to investigate the relationship between immune infiltration and the expression of core genes across all TCGA cancers. A p-value threshold of <0.05 was used to determine statistical significance, providing confidence in the observed associations while controlling for random variation. This analysis aimed to elucidate the role of key genes in the tumor immune microenvironment, providing insights into potential immunotherapy targets.

## 3 Results

### 3.1 Processing and screening of DEGs in the depression dataset

**FIGURE 1:**
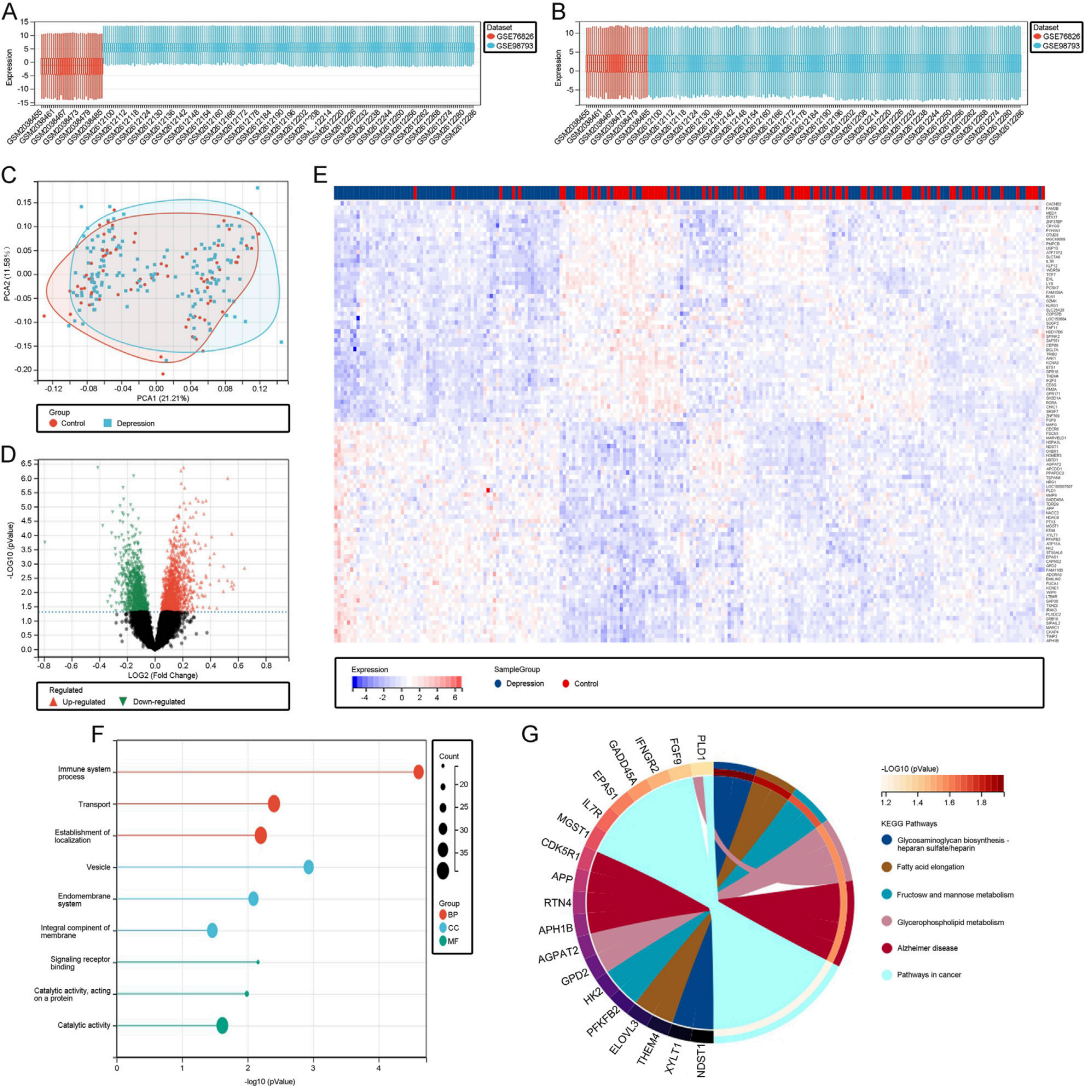
**(A)** Boxplot of the unnormalized raw data. **(B)** Boxplot of the data after normalization. **(C)** PCA plot illustrating the standardized depression and control datasets. **(D)** Volcano plot depicting differentially expressed genes (DEGs). **(E)** Heatmap of DEGs. **(F)** Gene Ontology (GO) analysis of DEGs. **(G)** KEGG pathway analysis of DEGs.

We identified two datasets, GSE76826 and GSE98793, from the GEO database. A box plot showed significant expression differences between the two datasets before normalization (Figure 1A). After normalization, the datasets were standardized, allowing for consistent processing and subsequent analysis (Figure 1B). PCA results further demonstrated that the datasets overlapped significantly after normalization, justifying their combined analysis (Figure 1C). Based on a threshold of P < 0.05 and |log2(FC)| > 0.5, we identified 111 DEGs. These DEGs were visualized using volcano plots (Figure 1D), which highlight genes with statistically significant expression changes, aiding in the identification of key upregulated and downregulated genes. Additionally, heatmaps (Figure 1E) displayed expression patterns across samples, emphasizing differential expression trends between depression and control groups, thus supporting the reliability of our DEG selection process. Functional enrichment analysis of these DEGs revealed that biological processes (BP) were primarily enriched in transport, establishment of localization, and immune system processes. Molecular functions (MF) were enriched in catalytic activity, acting on proteins, and signal transducer activity. Cellular components (CC) were enriched in membrane, vesicle, and organelle membrane components (Figure 1F). KEGG analysis highlighted pathways such as glycosaminoglycan biosynthesis, which plays a pivotal role in extracellular matrix organization and cellular communication. Glycosaminoglycans, such as heparan sulfate and chondroitin sulfate, are essential for modulating signaling pathways involved in neuroinflammation and neuronal repair. Dysregulation of these pathways has been implicated in the pathogenesis of depression, particularly in relation to chronic inflammation and impaired neuroplasticity. Similarly, glycerophospholipid metabolism, which is crucial for maintaining cellular membrane integrity and signal transduction, was identified. Alterations in this pathway have been linked to disrupted neuronal signaling and cognitive dysfunction in depressive disorders. Additionally, immune-related pathways like heparan sulfate biosynthesis further underline the inflammatory processes associated with depression, suggesting these molecular mechanisms may serve as potential therapeutic targets (Figure 1G).

### 3.2 Identification of key module genes highly correlated with depression progression through WGCNA

**FIGURE 2:**
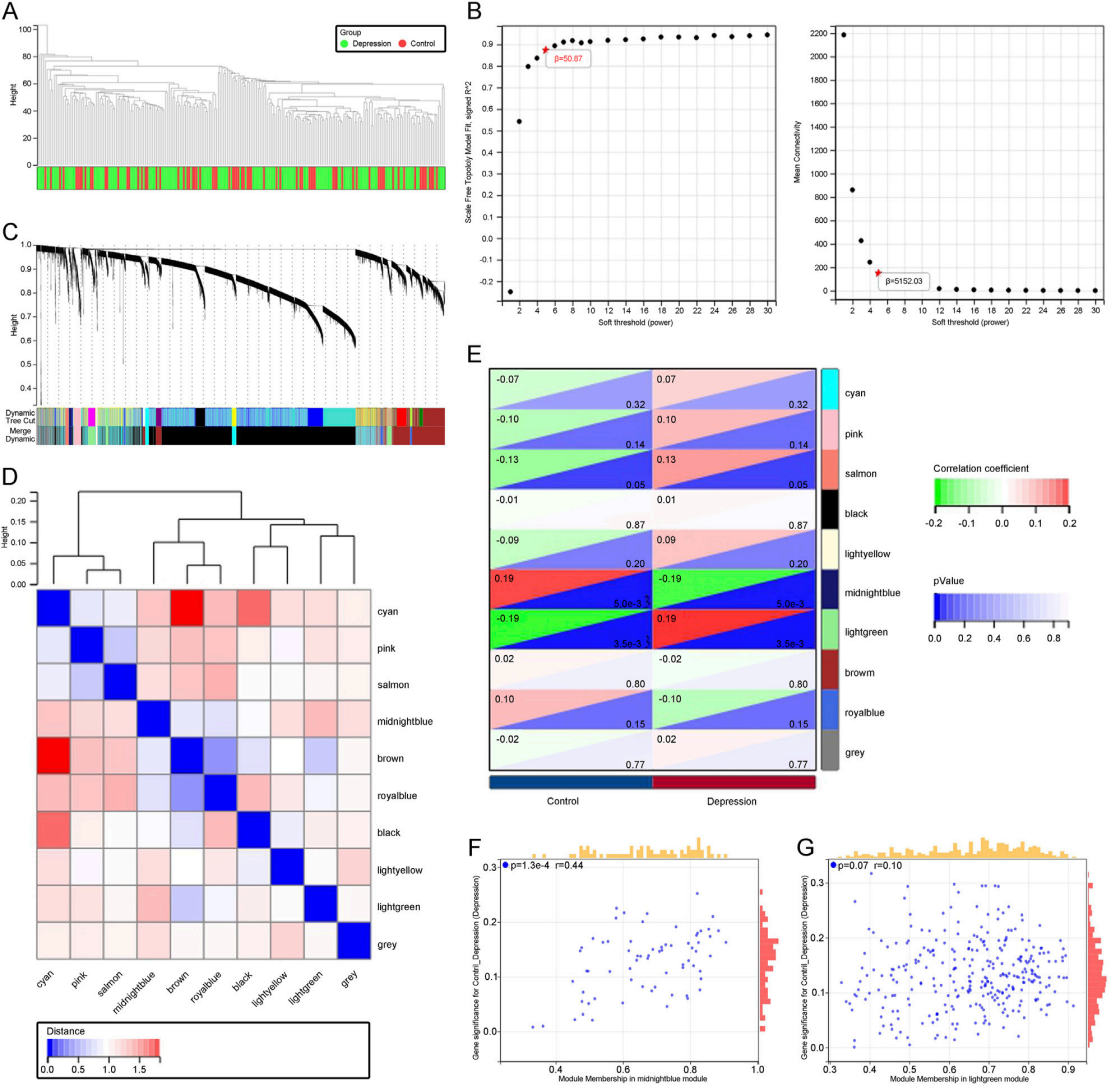
**(A)** Dendrogram illustrating sample clustering corresponding to individual samples. **(B)** Soft threshold (β = 5) with a scale-free topology fit index (*R* ^2^) = 0.87. **(C)** Original and merged modules displayed beneath the cluster tree. **(D)** Heatmap showing the co-expression of module eigengenes, with red indicating higher correlations and blue indicating lower correlations. **(E)** Dendrogram of module eigengene clustering. **(F)** Scatter plot depicting the relationship between module membership (MM) and gene significance (GS) in patients with depression. **(G)** Scatter plot showing the relationship between MM and GS in the control group.

To identify genes highly associated with depression, we performed WGCNA on the GSE76826 and GSE98793 datasets. By setting appropriate criteria, we removed samples that showed clear outliers in the sample clustering (Figure 2A). We then set the soft threshold to 5, with R2 > 0.87, to ensure a biologically meaningful scale-free network (Figure 2B). After merging modules with a high clustering cut-off of 0.25, ten modules were detected (Figure 2C). These modified and merged modules are displayed under the cluster tree. Subsequent examination of module correlations showed no significant inter-module correlations (Figure 2D). By analyzing the correlation between module eigengenes (ME) and clinical traits, we further explored the relationship between modules and clinical characteristics. Module eigengenes (ME) represent the first principal component of the gene expression profiles within a module and serve as a summary measure of the module’s overall expression. Correlation analysis between ME and clinical traits helps identify modules that are significantly associated with specific phenotypes, such as depression. In our analysis, The light green module was positively correlated with depression (r = 0.19, p = 3.5e-3), while the midnight blue module was negatively correlated with depression (r = −0.19, p = 5e-3; Figure 2E). Finally, in the scatter plots of MM and GS, the light green and midnight blue modules showed a strong correlation with depression (Figures 2F, G). Consequently, we further analyzed all genes within these two key modules. These modules provide insights into gene clusters that are functionally related to depression, potentially offering biomarkers or therapeutic targets for early diagnosis and treatment.

### 3.3 Identification of overlapping DEGs and WGCNA key module genes in depression through cross-analysis

**FIGURE 3:**
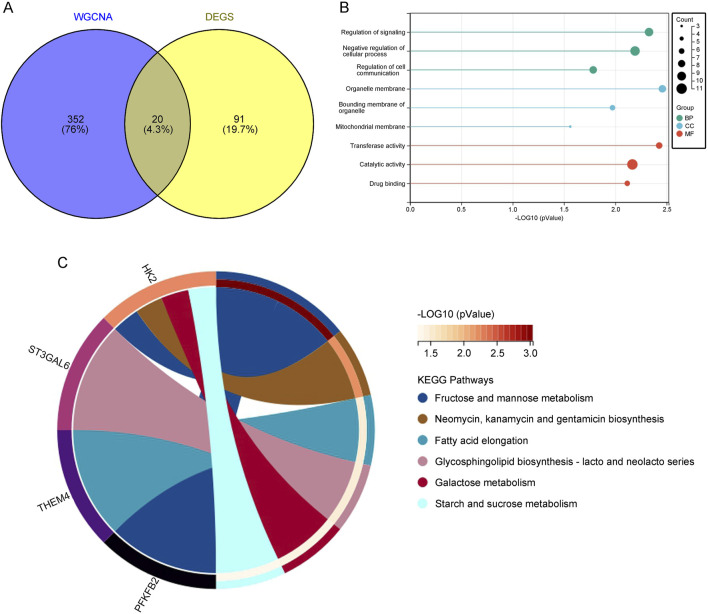
**(A)** Venn diagram displaying the overlap between key module genes and DEGs. **(B)** GO analysis of the overlapping genes. **(C)** KEGG pathway analysis of the overlapping genes.

To obtain biomarkers associated with depression, we used a Venn diagram to overlap DEGs obtained from the GEO database with key module genes identified from WGCNA, revealing 20 overlapping genes (Figure 3A). Functional enrichment analysis of these overlapping genes showed that, in the context of BP, they were mainly enriched in the negative regulation of cellular processes, regulation of signal transduction, and regulation of cell communication. MF was enriched in catalytic activity, transferase activity, and drug binding. CC was enriched in organelle membrane, organelle boundary membrane, and mitochondrial membrane (Figure 3B). KEGG analysis identified pathways such as fatty acid elongation and glycosphingolipid biosynthesis, which are essential for maintaining neuronal integrity and function, suggesting that dysregulation in these pathways might contribute to the pathogenesis of depression (Figure 3C).

### 3.4 Screening of core genes in depression using machine learning

**FIGURE 4:**
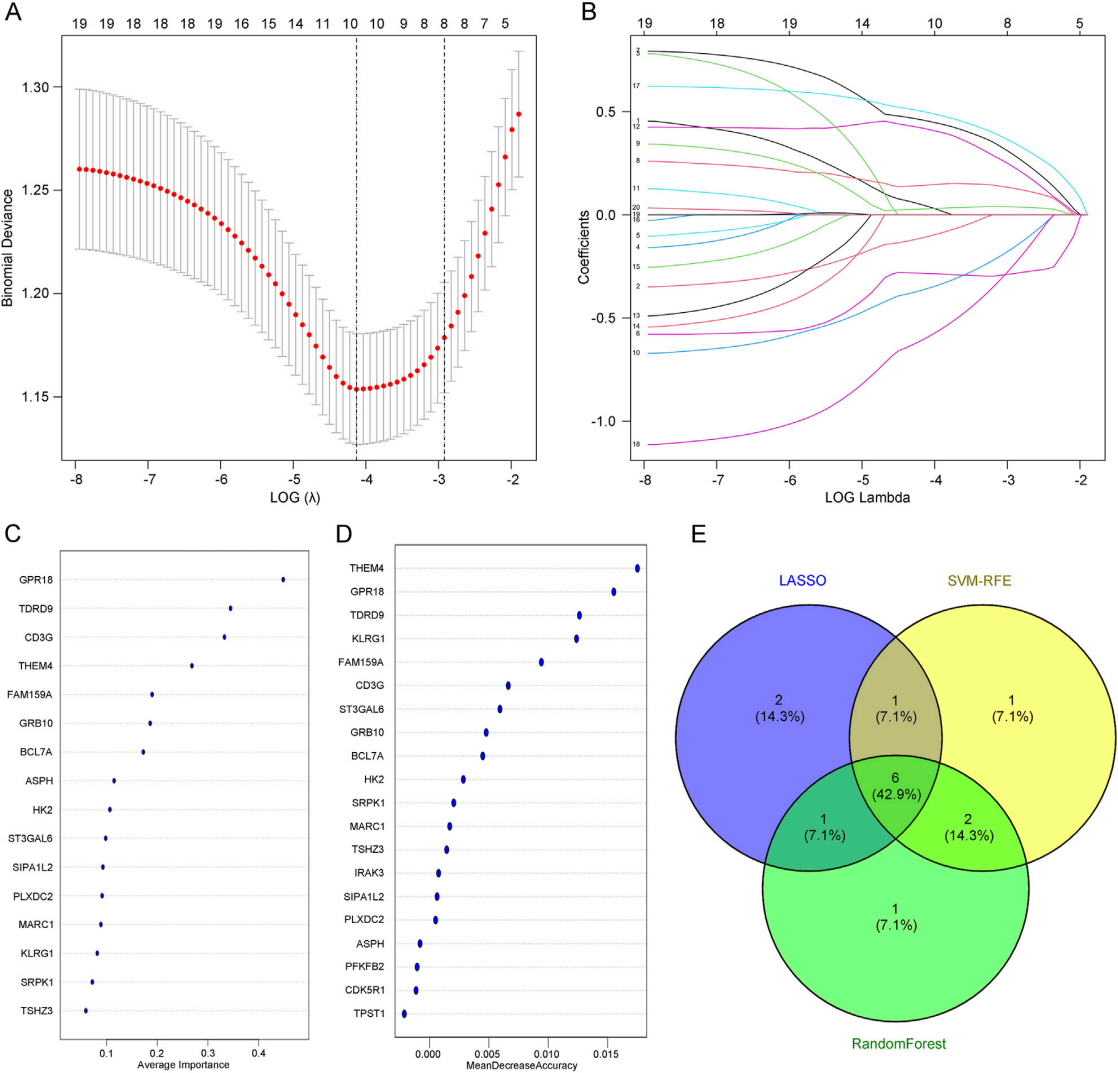
**(A)** LASSO regression analysis with adjusted minimum absolute shrinkage. **(B)** Feature selection within the LASSO regression model. **(C)** Selection and validation of biomarker signature gene expression using the SVM-RFE algorithm. **(D)** Selection and validation of biomarker signature gene expression using the RF algorithm. **(E)** Venn diagram illustrating gene selection across three different algorithms.

To screen for core genes, we combined three computational techniques to analyze the overlapping genes. LASSO regression analysis (Figures 4A, B) and SVM-RFE (Figure 4C) were employed to select core predictive genes from statistically significant univariate variables. Random forest with feature selection was used to calculate the correlation between error rate and the number of classification trees, followed by screening of core predictive genes (Figure 4D). Finally, we performed a cross-analysis using a Venn diagram of these three computational methods, identifying six core genes: GRB10, TDRD9, BCL7A, GPR18, KLRG1, and THEM4 (Figure 4E). The integration of these machine learning approaches ensures the robustness of gene selection, highlighting genes that may play critical roles in the molecular mechanisms underlying depression.

### 3.5 Validation of core gene expression in depression

**FIGURE 5:**
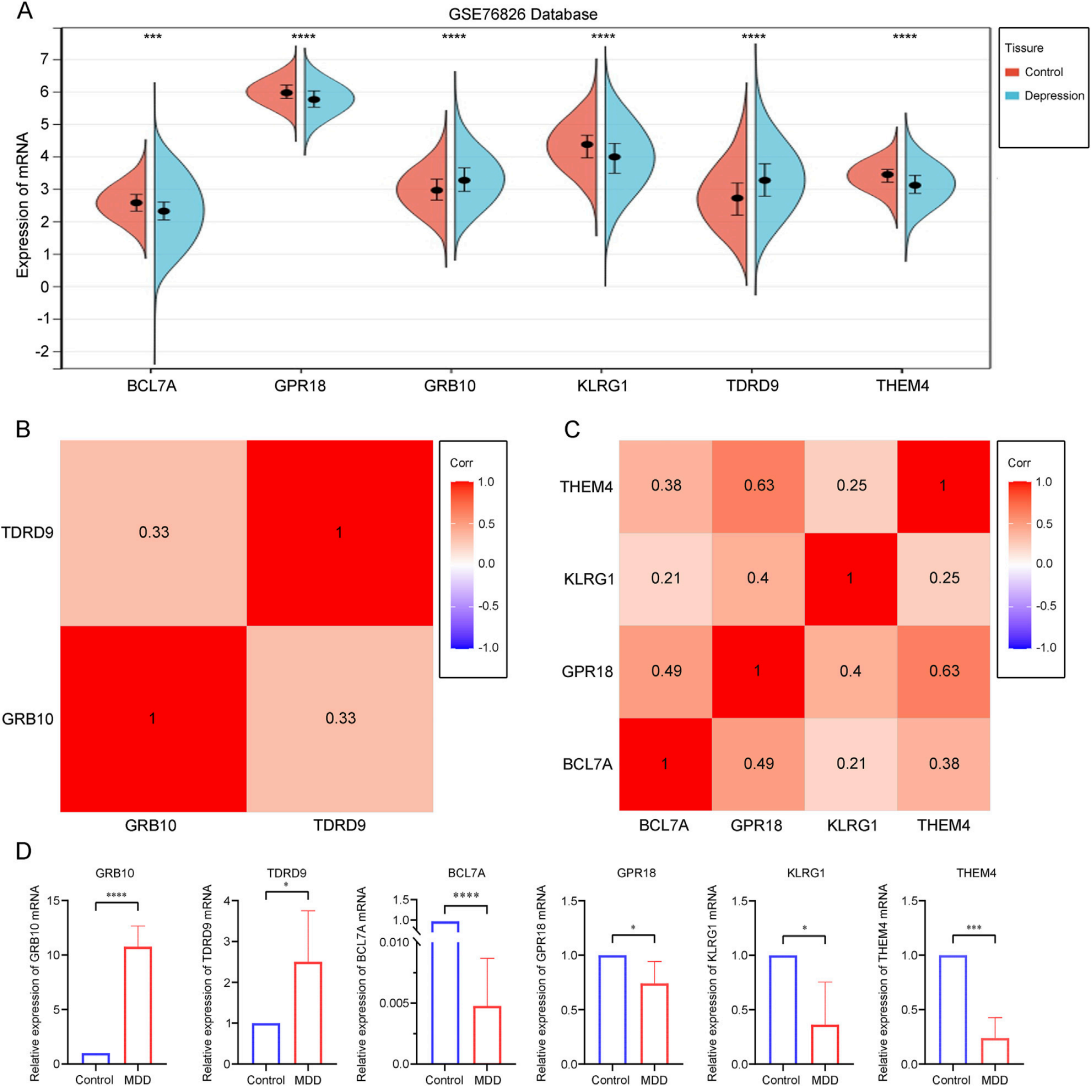
**(A)** Differential expression analysis of core genes within the dataset. **(B)** Correlation analysis of core genes with increased expression in depression. **(C)** Correlation analysis of core genes with decreased expression in depression. **(D)** A quantitative analysis of the mRNA transcription levels of core genes in the blood was conducted for both depressed patients (n = 6) and healthy controls (n = 8). Statistical analysis was performed using Student’s t-test, and significance levels are indicated as follows: p < 0.05 (*), p < 0.01 (), p < 0.001 (*), and p < 0.0001 ().

To ensure the accuracy of our results, we validated the expression of the six core genes in the GSE76826 dataset. The results showed that the expression levels of GRB10 and TDRD9 were significantly increased in depression patients compared to the control group, while the expression levels of BCL7A, GPR18, KLRG1, and THEM4 were significantly decreased, all with statistical significance (Figure 5A). Additionally, we explored the relationships among the upregulated and downregulated genes. GRB10 was positively correlated with TDRD9, suggesting functional similarities between these two genes (Figure 5B). Similarly, the positive correlations among BCL7A, GPR18, KLRG1, and THEM4 indicated shared functional characteristics (Figure 5C). Finally, we experimentally validated the expression of these six core genes in depression patients. qPCR results demonstrated that, compared to the normal control group, the expression of GRB10 and TDRD9 was significantly elevated in the blood of depression patients, while the expression of BCL7A, GPR18, KLRG1, and THEM4 was significantly reduced (Figure 5D). These findings are consistent with our previous results, confirming the crucial role of these six core genes in the progression of depression.

### 3.6 Gene Set Enrichment Analysis (GSEA) of core genes in depression

To gain a deeper understanding of the functions of core genes in depression, we performed a Gene Set Enrichment Analysis (GSEA) on these genes. First, we divided the depression tissue samples into two groups based on the median expression levels of core genes. The high BCL7A subgroup was enriched in pathways related to DNA replication, mismatch repair, primary immunodeficiency, and the low BCL7A subgroup was enriched in pathways such as Rig-I-like receptor signaling and the renin-angiotensin system (Supplementary Figure S1A). These findings suggest that BCL7A may contribute to maintaining genomic stability and immune regulation, processes that could be disrupted in depression. The low GPR18 subgroup showed enrichment in amino sugar and nucleotide metabolism, fructose and mannose metabolism, alanine metabolism, pantothenate and CoA biosynthesis, and folate biosynthesis (Supplementary Figure S1B). The involvement of GPR18 in metabolic pathways emphasizes its role in cellular energy homeostasis and neurotransmitter biosynthesis, which are critical for brain function and may be impaired in depressive disorders. The high GRB10 subgroup was enriched in aminoacyl-tRNA biosynthesis, DNA replication, primary immunodeficiency, and mismatch repair, whereas the low GRB10 subgroup was enriched in sulfur metabolism (Supplementary Figure S1C). These pathways highlight GRB10’s potential role in protein synthesis and stress response mechanisms. The high KLRG1 subgroup showed enrichment in folate biosynthesis, phenylalanine metabolism, and riboflavin metabolism, while the low KLRG1 subgroup was enriched in homologous recombination, primary immunodeficiency, and steroid biosynthesis (Supplementary Figure S1D). The high TDRD9 subgroup was significantly enriched in DNA replication, ribosome, and RNA polymerase, whereas the low TDRD9 subgroup was enriched in type II diabetes, folate biosynthesis, and sulfur metabolism (Supplementary Figure S1E). The high THEM4 subgroup was enriched in steroid biosynthesis, while the low THEM4 subgroup was enriched in complement and coagulation cascades, fructose and mannose metabolism, folate biosynthesis, and pantothenate and CoA biosynthesis (Supplementary Figure S1F). These results suggest that core genes regulate pathways involved in immune responses, energy metabolism, and cellular repair, all of which are closely linked to the pathogenesis of depression.

### 3.7 Interaction analysis of core genes in depression

**FIGURE 6:**
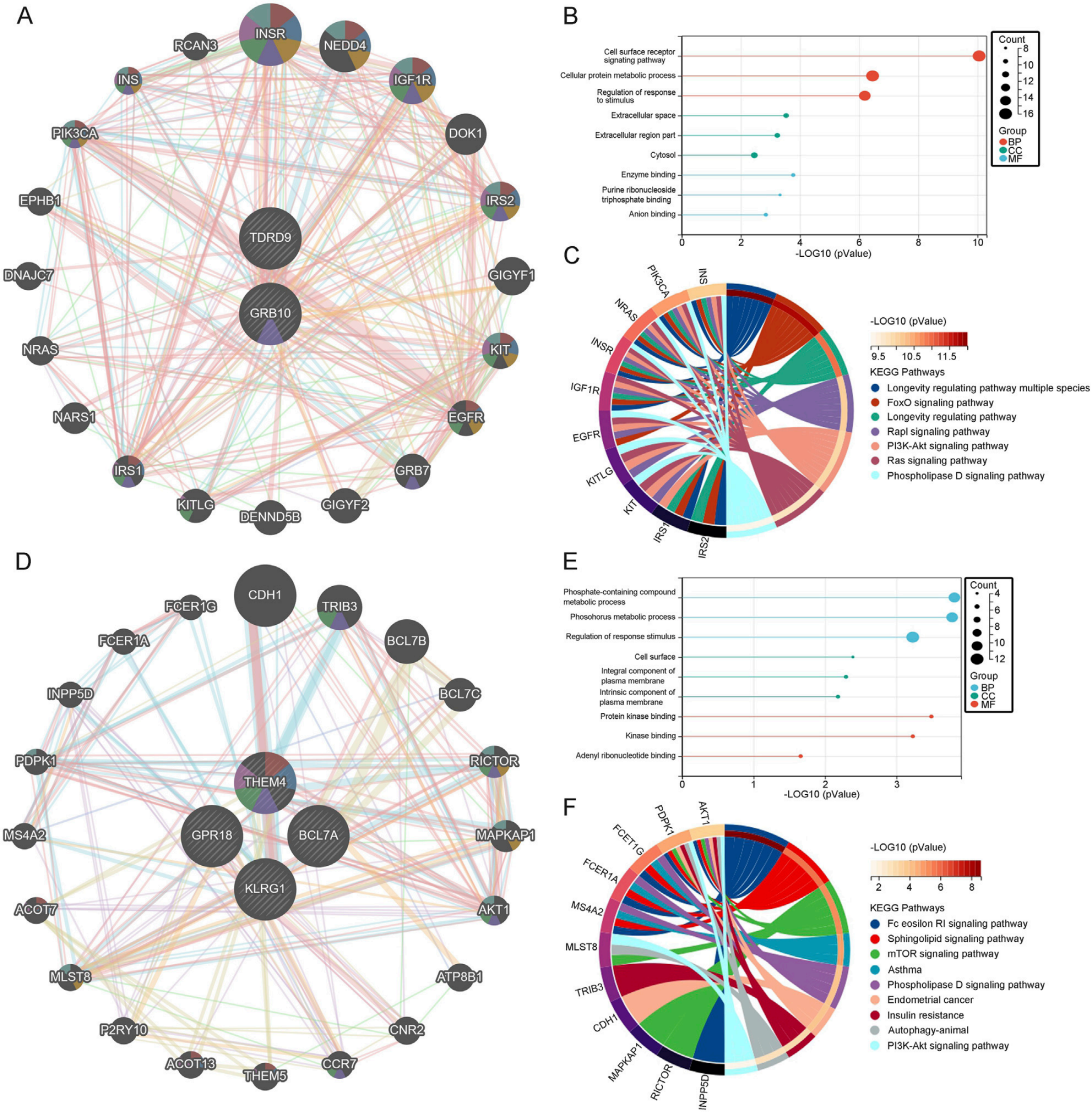
**(A)** Co-expression network of upregulated genes. **(B)** GO analysis of upregulated genes. **(C)** KEGG pathway analysis of upregulated genes. **(D)** Co-expression network of downregulated genes. **(E)** GO analysis of downregulated genes. **(F)** KEGG pathway analysis of downregulated genes.

To further explore the functions of core genes, we utilized the GeneMania database to identify the 20 most closely related genes to GRB10 and TDRD9, constructing a co-expression network (Figure 6A). GO/KEGG analysis of these genes revealed that biological process (BP) enrichment was mainly associated with cell surface receptor signaling pathways, cellular protein metabolic processes, and response to stimuli regulation. Molecular function (MF) enrichment was primarily related to enzyme binding, anion binding, and purine ribonucleotide binding. Cellular component (CC) enrichment was mainly associated with the cytoplasm, extracellular space, and extracellular region (Figure 6B). KEGG analysis identified significant pathways including the PI3K-Akt signaling pathway, FoxO signaling pathway, Rap1 signaling pathway, Ras signaling pathway, longevity regulating pathway, multi-species longevity regulating pathway, and phospholipase D signaling pathway (Figure 6C). These pathways indicate that GRB10 and TDRD9 are involved in critical cellular processes such as survival signaling, stress adaptation, and metabolic regulation, which may play central roles in the development and progression of depression. Using the same method, we identified the 20 most closely related genes to BCL7A, GPR18, KLRG1, and THEM4, constructing a co-expression network (Figure 6D). Enrichment analysis showed that BP enrichment was mainly associated with regulation of response to stimuli, phosphorus-containing compound metabolism, and phosphorus metabolism. MF enrichment was primarily related to protein kinase binding, kinase binding, and adenosine ribonucleotide binding. CC enrichment was mainly associated with components of the plasma membrane, intrinsic components of the plasma membrane, and the cell surface (Figure 6E). KEGG analysis revealed pathways such as Fc epsilon RI signaling pathway, sphingolipid signaling pathway, mTOR signaling pathway, asthma, phospholipase D signaling pathway, endometrial cancer, insulin resistance, fatty acid elongation, and autophagy in animals (Figure 6F). These pathways suggest that BCL7A, GPR18, KLRG1, and THEM4 play roles in immune signaling and metabolic pathways, further underscoring their relevance to depression.

### 3.8 Prognostic model for depression based on core genes

**FIGURE 7:**
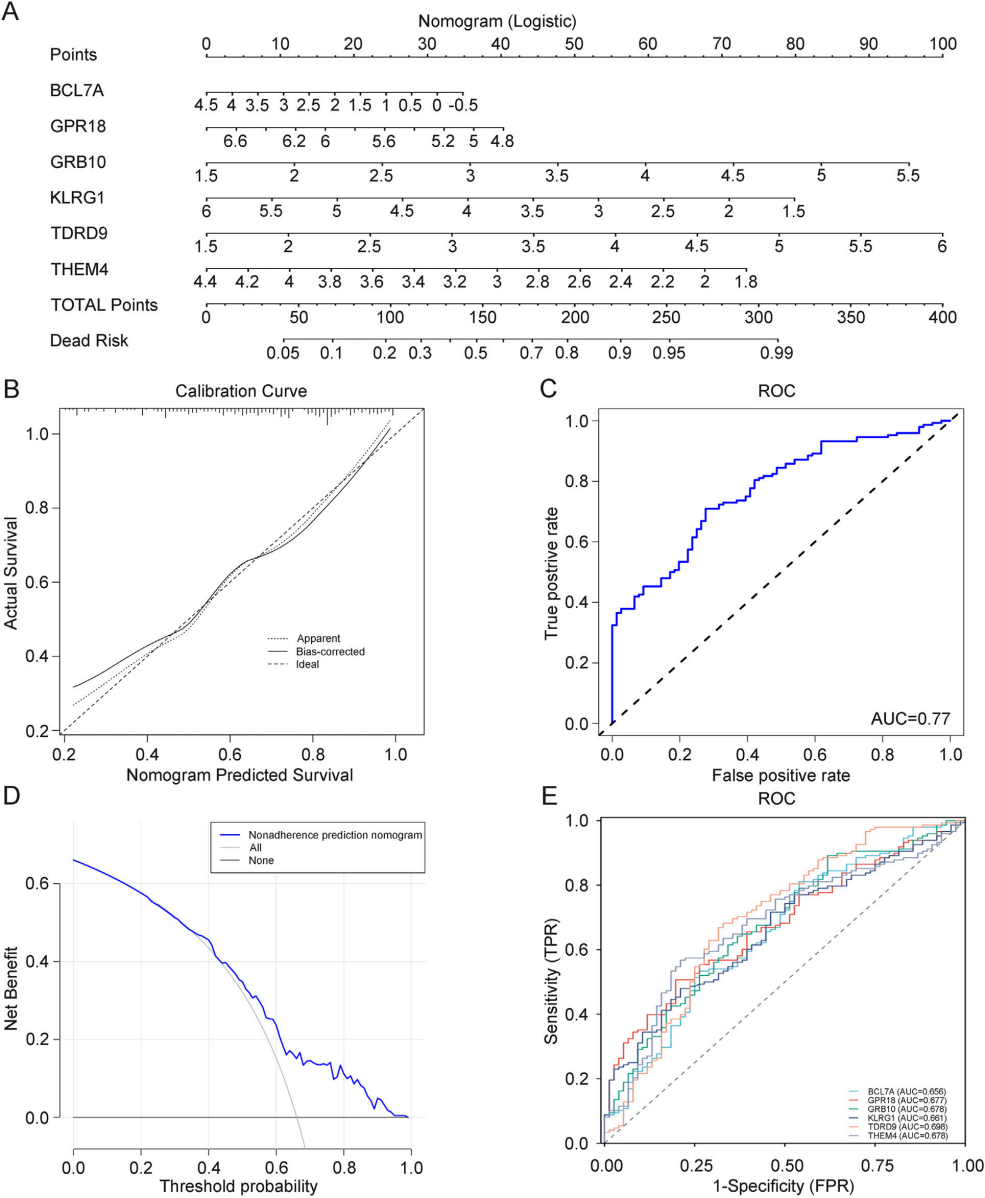
**(A)** Prognostic model for depression diagnosis. **(B)** Calibration curve evaluating the predictive accuracy of the prognostic model. **(C)** ROC curve assessing the clinical utility of the depression model. **(D)** DCA curve assessing the clinical utility of the depression model. **(E)** ROC curve for core genes.

To more accurately predict and assess the progression of depression, we constructed a nomogram model for depression diagnosis based on the core genes (GRB10, TDRD9, BCL7A, GPR18, KLRG1, and THEM4) using the rms package (Figure 7A). Calibration curves demonstrated minimal differences between actual and predicted risks, indicating that the depression nomogram model has good predictive capability (Figure 7B). Decision curve analysis (DCA) (Figure 7C) and ROC analysis (AUC: 0.77) (Figure 7D) further confirmed the strong predictive ability of this prognostic model. Finally, to determine the diagnostic utility of BCL7A, GPR18, GRB10, KLRG1, TDRD9, and THEM4, we performed ROC analysis on these six core genes. The results indicated that BCL7A (AUC: 0.656), GPR18 (AUC: 0.9677), GRB10 (AUC: 0.678), KLRG1 (AUC: 0.661), TDRD9 (AUC: 0.698), and THEM4 (AUC: 0.678) exhibit high diagnostic value for depression (Figure 7E). This model provides a robust tool for identifying individuals at risk of depression, with potential for integration into clinical workflows to guide early intervention and personalized treatment strategies.

To investigate whether these depression-related findings have broader implications, we extended our analysis to explore the expression and functional relevance of the six identified core genes in pan-cancer datasets. This transition builds on the potential overlap of molecular pathways, such as immune regulation and metabolic processes, between depression and cancer. By analyzing core gene expression across multiple cancer types, we aimed to uncover their broader biological roles and potential clinical applications.

### 3.9 Expression of core genes in pan-cancer

To investigate the correlation between cancer and depression, we examined the expression of BCL7A, GPR18, GRB10, KLRG1, TDRD9, and THEM4 across various cancer types using the TCGA database. The results showed significant differences in the expression of these six core genes in pan-cancer compared to normal tissues, and these differences were statistically significant (Supplementary Figure S2A). The altered expression patterns suggest that these genes may play distinct roles in tumorigenesis and tumor progression, potentially acting as oncogenes or tumor suppressors depending on the context. For example, overexpression of GRB10 and TDRD9 may enhance cancer cell proliferation, while downregulation of BCL7A, GPR18, KLRG1, and THEM4 could contribute to reduced immune surveillance and tumor evasion. To validate these findings, we combined data from the GTEx database with TCGA data, confirming significant and statistically meaningful differences in the expression of these six core genes in pan-cancer (Supplementary Figure S2B). Therefore, these six core genes exhibit potential as cancer biomarkers or therapeutic targets, offering new perspectives and directions for precision medicine in cancer treatment.

### 3.10 Prognostic value of core genes in pan-cancer and immune infiltration analysis

**FIGURE 8:**
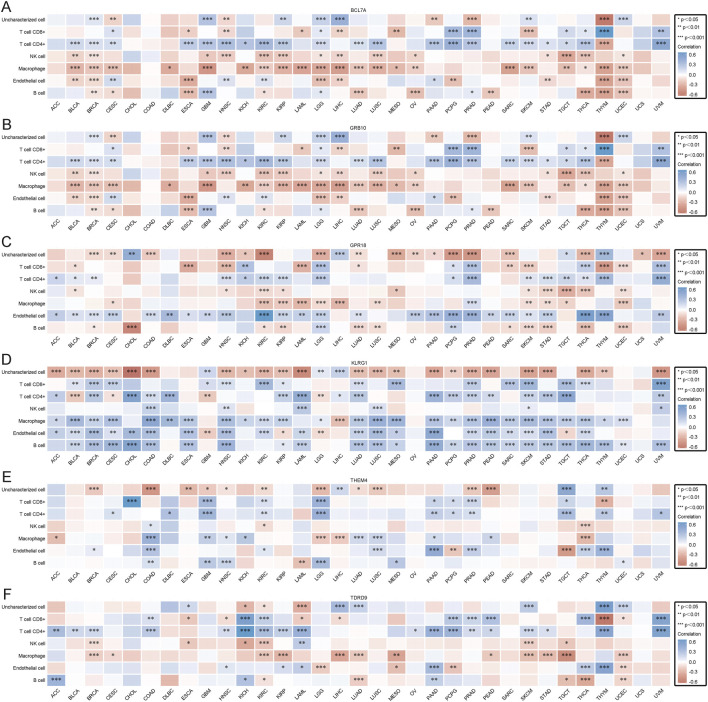
**(A)** EPIC immune scores for BCL7A. **(B)** EPIC immune scores for GPR18. **(C)** EPIC immune scores for GRB10. **(D)** EPIC immune scores for KLRG1. **(E)** EPIC immune scores for TDRD9. **(F)** EPIC immune scores for THEM4.

To further understand the impact of core gene expression on cancer patient prognosis, we analyzed the relationships between core gene expression and overall survival (OS) (Supplementary Figure S3A), disease-specific survival (DSS) (Supplementary Figure S3B), and progression-free survival (PFS) (Supplementary Figure S3C) in pan-cancer patients. Prognostic analysis of 38 cancer types revealed that different genes exhibit protective and risk factor states across various cancers. For instance, BCL7A and GRB10 showed protective roles in some cancers, possibly through mechanisms such as enhancing immune infiltration or suppressing oncogenic pathways, while THEM4, TDRD9, and KLRG1 acted as risk factors in others, potentially by inhibiting tumor suppressor functions. This provides robust support for understanding their roles and predictive value in cancer and informs clinical research and treatment decisions. Subsequently, we calculated the correlation between core gene expression and immune cell infiltration in various cancers using the EPIC algorithm to better understand the role of core genes in tumor immune responses. Results indicated that core genes can influence cancer progression by regulating immune cells, with BCL7A and GRB10 showing positive correlations with immune cell infiltration in most cancers (Figures 8A, B). These genes may enhance anti-tumor immunity by recruiting and activating immune cells, such as T cells and macrophages, in the tumor microenvironment. Conversely, KLRG1, THEM4, and TDRD9 exhibited negative correlations with immune cell infiltration (Figures 8D–F). Their downregulation may suppress immune activation, allowing tumors to evade immune surveillance. The correlation between GPR18 expression and immune cell infiltration in cancer is more complex, showing both positive and negative correlations depending on the cancer type (Figure 8C). This context-dependent effect of GPR18 highlights its multifaceted role in modulating immune responses, which may vary depending on the tumor microenvironment. These findings suggest that core gene expression not only correlates with patient prognosis but also significantly affects tumor immune responses. Genes such as BCL7A and GRB10 may exert anti-cancer effects by promoting immune cell infiltration, whereas KLRG1, THEM4, and TDRD9 might suppress immune cell infiltration, facilitating tumor progression. The varied impact of GPR18 highlights the complexity of immune modulation in cancer, suggesting its role may differ across tumor types. These insights deepen our understanding of the mechanisms of core genes in cancer and could provide important molecular targets and theoretical foundations for developing new cancer immunotherapies and personalized treatment strategies.

Overall, these findings provide novel insights into the dual and context-specific roles of core genes in cancer immunity and progression. By linking immune infiltration with gene expression patterns, our study offers a deeper understanding of shared mechanisms between depression and cancer. This understanding lays the groundwork for targeted immunotherapies and precision medicine approaches that address overlapping pathways in these diseases.

## 4 Discussion

Depression is currently the most prevalent chronic mental disorder (Zeng et al., 2020). Some of the primary symptoms include depression, anorexia, low self-esteem, pain, and sleep disturbances (Nishiguchi et al., 2021). Patients with prolonged depression are at a higher risk of developing suicidal thoughts and engaging in suicidal behavior (Xia et al., 2023). Clinical data indicate that more than 300 million people worldwide suffer from depression (Xie et al., 2020). By 2030, depression is expected to surpass all other health-related conditions as the leading cause of disability (Chu et al., 2019). Currently, pharmacotherapy remains the primary treatment for depression (Farah et al., 2016). Although various antidepressant medications are available, they are associated with significant side effects, and to date, no drug has been developed specifically to target depression (Bian et al., 2022). Therefore, our current focus is on identifying precise targets for the accurate treatment of depression.

In recent years, the rapid development of bioinformatics has played a crucial role in the medical field. Through functional enrichment analysis of molecules, we can systematically and comprehensively study the onset and progression of diseases (Liang et al., 2019; Gómez-López and Valencia, 2008). Gene screening has also increasingly incorporated machine learning algorithms and WGCNA (Langfelder and Horvath, 2008). However, there remains a lack of literature utilizing bioinformatics to study depression.

In light of this, we developed a comprehensive and systematic evaluation framework for depression. By integrating WGCNA, machine learning algorithms, and PPI analysis, our study uniquely bridges bioinformatics with advanced computational techniques, enabling the identification of shared genetic mechanisms between depression and cancer. This integrative approach represents a significant advancement in understanding the molecular underpinnings of these complex conditions. Using this framework, combined with WGCNA, machine learning algorithms, and PPI analysis, we examined the core genes and molecular pathways in patients with depression, leading to significant advancements in the treatment of this disorder.

In this study, we identified a total of 111 DEGs. WGCNA analysis revealed 372 core genes and 10 modules. Cross-analysis identified 20 overlapping genes, and machine learning techniques further pinpointed six core genes. Among them, GRB10 and TDRD9 were significantly upregulated, while BCL7A, GPR18, KLRG1, and THEM4 were significantly downregulated in patients with depression. These findings provide novel insights into the shared molecular mechanisms between depression and cancer, particularly through their roles in immune regulation and metabolic pathways. The identified core genes may serve as dual-purpose biomarkers or therapeutic targets, offering a foundation for precision medicine approaches that address overlapping pathways in both diseases. We constructed a bar graph model for depression diagnosis, confirming the diagnostic value of these six core genes, indicating their considerable significance in depression. Subsequent ROC analysis demonstrated that all core genes are crucial in depression, suggesting their potential diagnostic relevance in therapeutic treatment. Finally, we validated the expression trends of these six core genes in the blood of depressed patients using qPCR, finding that the trends were consistent with our results compared to the control group. This comprehensive analysis underscores the translational potential of our findings, paving the way for future research into targeted therapies for depression and cancer.

Interestingly, some studies have proposed a potential link between depression and cancer (Brewer, 2008; Leis et al., 2022; Bieliauskas and Garron, 1982). However, there is no recent research precisely exploring the connection between these two conditions. Therefore, we investigated the significance of depression-related core genes in pan-cancer.

Our findings revealed that these core genes play significant roles in cancer. However, the expression trends of these genes in depression are not consistent across all cancers, as observed in the TCGA dataset. For example, GRB10 and TDRD9, which are upregulated in depression, display variable expression patterns in different cancer types, reflecting their context-dependent roles. Similarly, BCL7A, GPR18, KLRG1, and THEM4, which are downregulated in depression, also exhibit diverse expression trends across cancers. This variability suggests that these genes may serve dual roles, functioning as either protective or risk factors depending on the specific cancer type and its associated molecular mechanisms. For instance, BCL7A and GRB10, which were found to be protective factors in certain cancers, are known to regulate immune-related pathways and cellular signaling, potentially enhancing anti-tumor immunity and suppressing oncogenic activity. Conversely, THEM4, TDRD9, and KLRG1, which act as risk factors in some cancers, might contribute to tumor progression through immune evasion mechanisms or by impairing apoptosis pathways. GPR18, with its dual and context-dependent roles, is involved in immune cell recruitment and modulation, reflecting its complex contribution to tumor immune microenvironments. This variability emphasizes the importance of context and tissue specificity when investigating the shared mechanisms between depression and cancer. Additionally, immune infiltration results indicate that core genes may influence the occurrence and development of cancer through immune infiltration. These findings suggest that these genes serve as key regulators in both diseases by modulating shared mechanisms, such as immune dysregulation and chronic inflammation. While the observed differential expression trends between depression and cancers highlight the complexity of these genes’ roles, they also present opportunities for further research to elucidate their context-specific functions and therapeutic potential.

In summary, this study made substantial progress by systematically analyzing the core genes and molecular pathways in depression patients, using a comprehensive evaluation framework that incorporates WGCNA, machine learning algorithms, and PPI analysis. We identified six core genes (BCL7A, GPR18, GRB10, KLRG1, TDRD9, and THEM4), confirming their critical value in diagnosing depression. Additionally, we preliminarily explored the roles of these core genes in pan-cancer, discovering their significant impact on cancer development and progression. However, this study has some limitations. First, although we validated the expression trends of core genes in the blood of depressed patients using qPCR, the sample size was relatively small, necessitating further validation with larger samples in future studies. Second, while our analysis provides valuable insights into gene expression, it does not address the functional roles of these genes. Functional studies, such as gene knockdown or overexpression experiments, will be crucial to uncover the precise molecular mechanisms underlying their roles in depression and cancer. Third, this research primarily focused on gene expression, leaving the complex pathological mechanisms of depression needing further exploration. To address these gaps, single-cell sequencing could be employed to dissect cell-type-specific expression patterns of the six core genes in both depression and cancer tissues, enabling the identification of specific cell populations or microenvironments where these genes are active. For instance, single-cell RNA sequencing of tumor tissues from patients with co-morbid depression could help elucidate how immune cell populations or stromal cells express these genes, shedding light on their dual roles in both conditions. Similarly, proteomics approaches could validate the functional relevance of these genes at the protein level. Proteomic profiling of plasma or cerebrospinal fluid (CSF) could reveal post-translational modifications or interactions of these proteins, providing further evidence of their involvement in key biological pathways. Finally, given the observed expression variability across cancers, future research should focus on identifying context-specific regulatory mechanisms, such as transcription factor binding or epigenetic modifications, that drive these differential expression patterns. Integrative approaches combining transcriptomics, epigenomics, and proteomics data could provide a more comprehensive understanding of the shared and distinct roles of these genes in depression and cancer. These studies would pave the way for tailored therapeutic strategies targeting overlapping pathways in both diseases.

## 5 Conclusion

This study conducted a comprehensive bioinformatics analysis, identifying six core genes (BCL7A, GPR18, GRB10, KLRG1, TDRD9, and THEM4) associated with depression and validating their diagnostic value in the context of the disorder. Additionally, we preliminarily investigated the roles of these core genes in pan-cancer, revealing their impact on cancer occurrence and progression. These findings highlight the potential clinical relevance of the identified core genes, which could serve as both diagnostic biomarkers and therapeutic targets. By addressing shared mechanisms in depression and cancer, this research provides a foundation for the development of targeted treatments and precision medicine approaches. Such efforts are essential for advancing effective strategies for the treatment and prevention of depression, and for exploring novel interventions in cancer therapy.

## Data Availability

The original contributions presented in the study are included in the article/Supplementary Material, further inquiries can be directed to the corresponding authors.
