# Supplementary material for: Depression-related innate immune genes and pan-cancer gene analysis and validation
Source: Front Genet. 2025 Jan 10;15:1521238. doi: 10.3389/fgene.2024.1521238 (PMC11757255; doi:10.3389/fgene.2024.1521238)
Supplement: Supplementary file 1 [file DataSheet1.pdf]

Supplementary Material

GEO Data set	Platform	Depression	Control
GSE76826	GPL6947	20	12
GSE98793	GPL570	128	64

Supplementary Table 1. Information obtained from the microarray dataset.

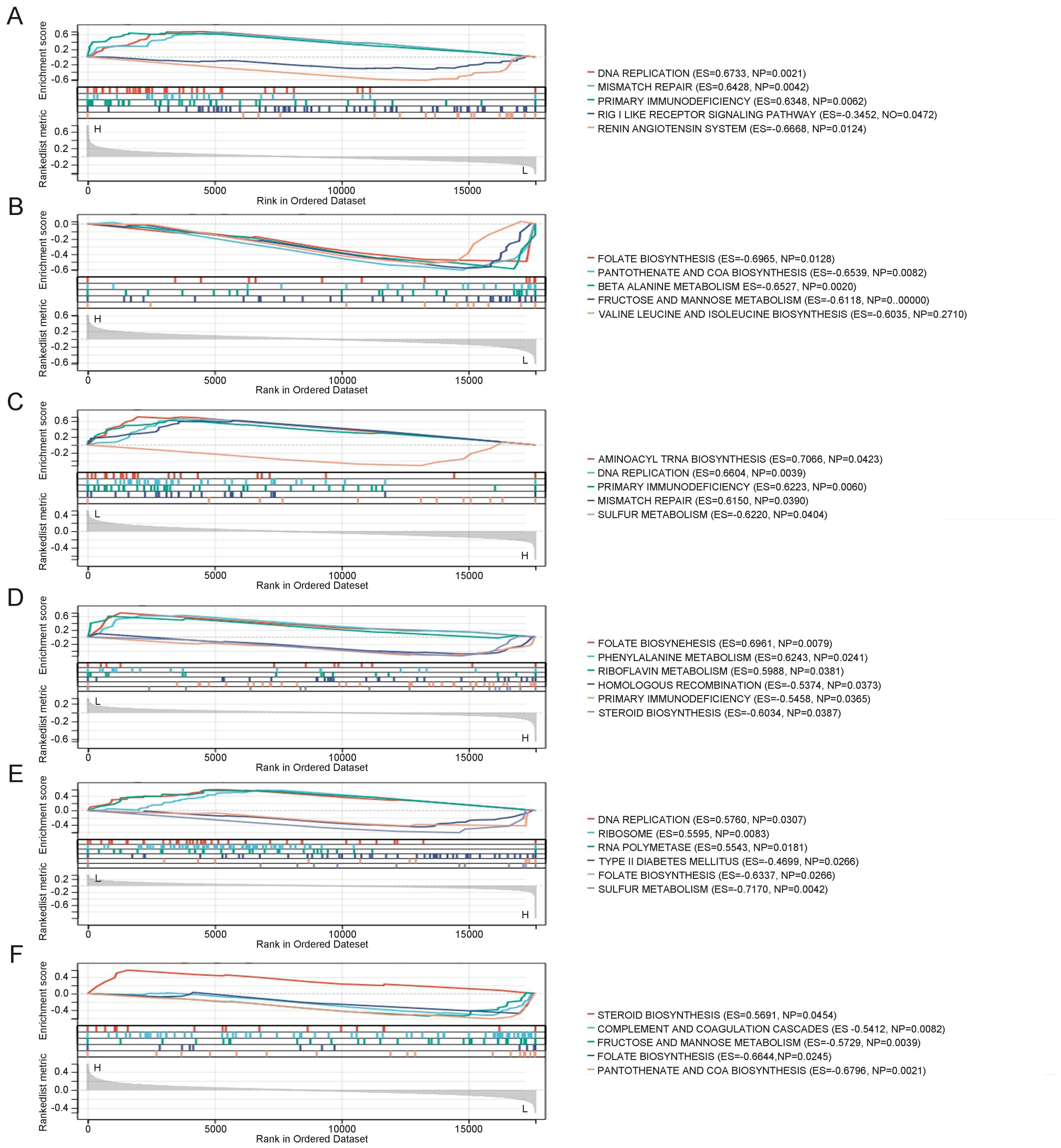

Supplementary Figure 1: (A) Gene Set Enrichment Analysis (GSEA) for BCL7A. (B) GSEA for GPR18. (C) GSEA for GRB10. (D) GSEA for KLRG1. (E) GSEA for TDRD9. (F) GSEA for THEM4.

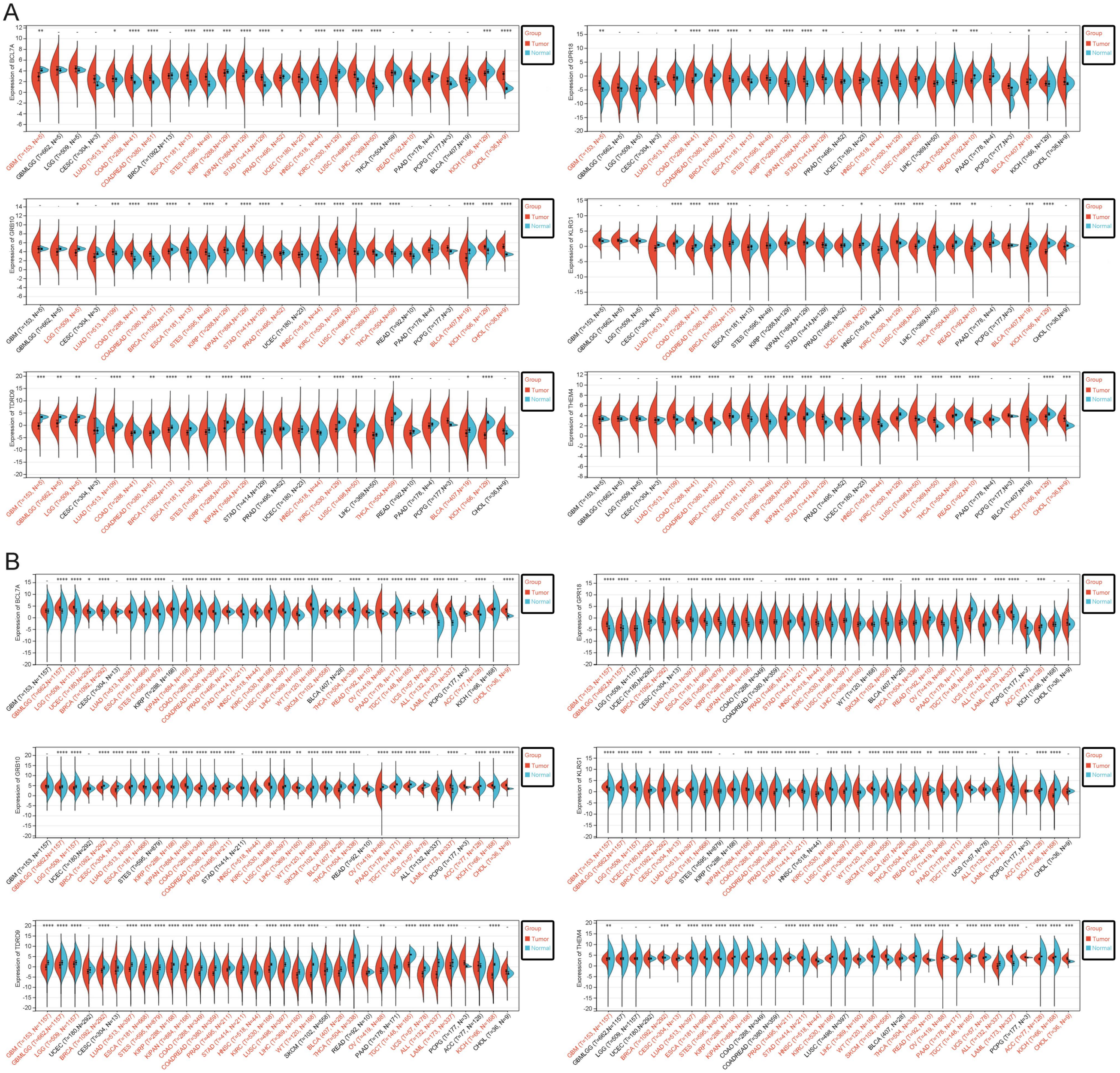

Supplementary Figure 2: (A) Expression levels of core genes in pan-cancer patients from the TCGA dataset. (B) Expression levels of core genes in pan-cancer patients from the TCGA and GTEx datasets.

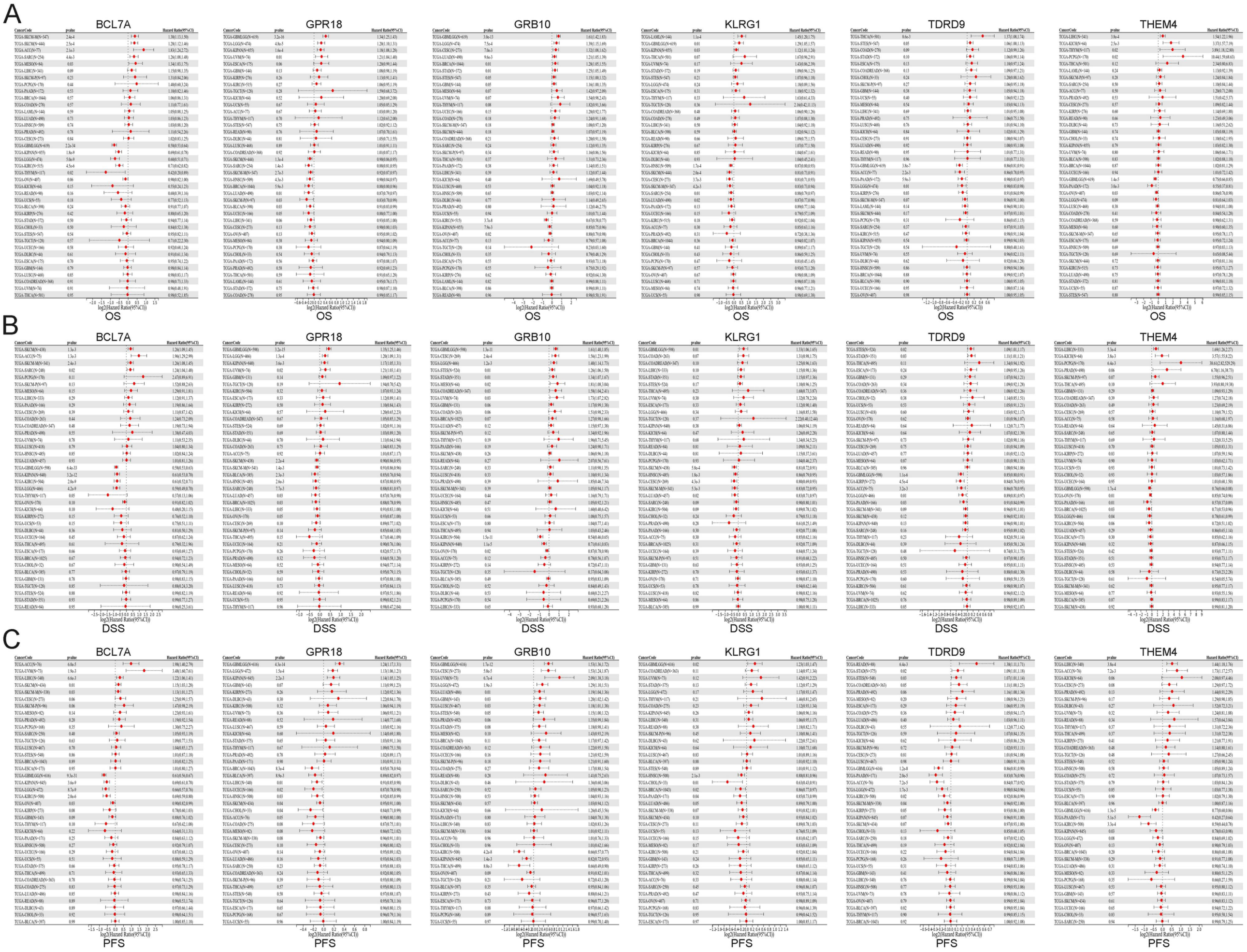

Supplementary Figure 3: (A) Overall survival (OS) prognosis analysis of core genes across pan-cancer datasets. (B) Disease-specific survival (DSS) prognosis analysis of core genes across pan-cancer datasets. (C) Progression-free survival (PFS) prognosis analysis of core genes across pan-cancer datasets.
